# Sex differences in vascular endothelial function in predicting cardiovascular events in hypertensive patients

**DOI:** 10.1007/s11739-026-04311-3

**Published:** 2026-04-17

**Authors:** Maria Perticone, Raffaele Maio, Edoardo Suraci, Elena Succurro, Francesco Andreozzi, Francesco Perticone

**Affiliations:** 1 Department of Medical and Surgical Sciences, University Magna Græcia of Catanzaro, Viale Europa, 88100 Catanzaro, Italy; 2 Geriatric Unit, Azienda Ospedaliero Universitaria R. Dulbecco, Catanzaro, Italy; 3 Internal Medicine Unit, Azienda Ospedaliero Universitaria R. Dulbecco, Catanzaro, Italy

**Keywords:** Endothelial dysfunction, Cardiovascular events, Gender medicine, Atherosclerosis

## Abstract

The prevalence of cardiovascular morbidity and mortality is higher in females than in males, probably for different pathogenetic mechanisms operating in the onset and progression of atherosclerotic vascular disease. Endothelial dysfunction is also recognized as an important and independent predictor of cardiovascular events. Thus, we designed this study to detect possible differences in endothelial function between sexes and their effect on cardiovascular prognosis. We enrolled 844 Caucasian hypertensives (451 males and 393 females, aged 49.5 + 10.9 years). Endothelial function was investigated by strain-gauge plethysmography. Compared with males, females showed a significantly lower endothelium-dependent vasodilation (acetylcholine-stimulated peak percent increase 268  ± 1113 vs 309 ± 108% increase from basal). During the follow-up period of 9.3 ± 3.2 years, 252 new fatal and non-fatal cardiovascular outcomes (3.20%) occurred: 151 coronary (1.92%), 61 cerebrovascular (0.77%), and 40 deaths (0.51%), with a higher incidence in females than in males [MACE (3.79 vs 2.68%; *P* = 0.001) and coronary events (2.24 vs 1.69%; *P* = 0.035), cerebrovascular events (0.93 vs 0.64%; *P* = 0.135) and all-cause mortality (0.63 vs 0.40%; *P* = 0.155)]. In multivariate Cox regression analysis, endothelial function resulted an independent predictor of MACE (HR = 0.82; 95% CI 0.73–0.952) and coronary events (HR = 0.81; 95% CI 0.69–0.95), together with hs-CRP, age, LDL-cholesterol and triglyceride in the whole population, and in females and males, separately. These results were also confirmed in ROC analysis, that demonstrated a different cut-off value of endothelial function between groups (275% for males, 214% for females). Our results confirm, in hypertensive patients, the existence of significant differences between sexes in the occurrence of cardiovascular events, probably attributable to a lower vasodilating property of vascular endothelium in females.

## Introduction

Cardiovascular diseases remain one of the most important causes of death worldwide, especially in high-income countries, also due to the constant increase in obesity and diabetes prevalence, as reported by the global burden of disease study [1]. Another important element emerged from the same report regarding cardiovascular mortality, which is higher in women and in the more advanced decades of life. These findings lead to a very important consideration, in particular the limited attention paid to the utility of both primary and secondary cardiovascular prevention.

Although, for many years, coronary heart disease was considered an exclusively male disease, it is the main cause of death in women too, even if there are different pathogenetic mechanisms between the two sexes that contribute to the onset and progression of atherosclerotic vascular disease. According to this, there is increasing evidence demonstrating that females, especially the younger ones, have a higher mortality rate after acute myocardial infarction despite pharmacological treatment or primary percutaneous coronary interventions [2–4]. A very partial explanation can be found in the fact that females’ heart and vasculature are smaller and more rigid than males’ [5]; furthermore, it is known that female coronary artery diameters appear to be smaller even after correction for body surface area [6].

For many years, increasing and strong evidence have demonstrated the primary role of endothelial dysfunction in the appearance and progression of atherosclerotic vascular disease [5–8], and our group was the first which demonstrated its prognostic role for new cardiovascular events in a group of never-treated hypertensives [9], successively confirmed in other settings of patients [10]. These results have found biological plausibility in the identification and understanding of new pathogenetic pathways involving the endothelium, allowing us to overcome the simple mechanistic concept of lining the vascular wall. In fact, the activation of endothelial cells represents a crucial step in atherosclerosis because they reduce their vasoprotective effects such as inhibition of platelet aggregation, suppression of adhesion of leukocytes and monocytes on the endothelial surface, and inhibition of migration and proliferation of vascular smooth muscle cells [7–10, 12].

As is widely known, endothelial dysfunction, initially investigated in hypertensive patients [7], also plays a very important role in the pathogenesis of hypertension itself by offsetting the balance between vasodilator and vasoconstrictor agents and in the regulation of systemic hemodynamics [13, 14]. In fact, as already reported in the past, the modifications of the vascular wall distensibility, induced by a lower bioavailability of nitric oxide, interfere with the type of shear stress—oscillatory vs steady laminar—that increases oxidative stress through the activation of the nuclear factor-kappa *B* [15–18]. All these mechanisms activate and perpetuate reverberating mechanisms that contribute to the onset and progression of atherosclerotic disease in different clinical conditions [7–10, 19]. Over the years, the interest in sex disparity increased, particularly with regard to risk factors and the related pathogenic mechanisms responsible for the onset and progression of organ damage. Interestingly, this sex-related difference seems to be evident also for endothelial function, as reported by some not conclusive studies [20–25]. Anyway, there is no evidence about possible differences between men and women, and whether this difference may differently impact the cardiovascular prognosis. On these bases, the aim of the present study was to evaluate: 1. possible differences in endothelial function between the two sexes, and 2. whether this difference may have a different cardiovascular prognostic impact.

## Materials and methods

For this population-based prospective study, we enrolled 844 newly diagnosed Caucasian hypertensive outpatients [451 men and 393 women, aged 30–72 years (mean ± SD = 49.5 ± 10.6)], participating in the CATanzaro metabolic risk factors (CATAMERI) study and referred to our University Hospital for the evaluation of their cardiovascular and/or metabolic risk profile. The CATAMERI study was approved on October 17th, 2012 (approval number 2012.63) by the Ethics Committee of the Azienda Ospedaliero-Universitaria Mater Domini of Catanzaro (Italy).

Inclusion criteria were age ≥ 30 years, new diagnosis of essential hypertension, and ability to sign informed consent to enter the study protocol. Exclusion criteria were: secondary forms of hypertension detected by a specific protocol; chronic kidney disease defined by serum creatinine value ≥ 1.5 mg/dL; type 2 diabetes mellitus defined as HbA1c ≥ 6.5%, or fasting plasma glucose ≥ 126 mg/dL, or use of antidiabetic drugs; heart failure defined by both clinical and echocardiographic findings; previous cerebral and cardiovascular events; cardiomyopathies; peripheral vascular diseases; rheumatic and non-rheumatic valvular heart disease or prosthetic valves; malignant diseases; and liver diseases.

All procedures were conducted according to the principles defined in the *Declaration of Helsinki*, and all participants gave their informed written consent to study participation.

### Data collection and measures

The demographic and clinical data were obtained at the first eligibility visit; on this occasion, all patients underwent review of their medical history, physical examination, and anthropometric evaluation: weight, height, and body mass index (BMI) expressed as *K*g/m^2^.

Laboratory tests were carried out after a fasting period of at least 12 h. Fasting plasma glucose was obtained by the glucose oxidase method (Glucose Analyzer, Beckman Coulter SpA, Milan, Italy), while plasma fasting insulin was measured in duplicate by a highly specific radioimmunoassay. Calculation of insulin resistance (IR) was obtained by the homeostasis model assessment (HOMA) from the fasting glucose and insulin concentrations according to the equation: HOMA = [insulin (μU/mL _*_ glucose (mmol/L)]/22.5 [26]. Lipid profile was obtained by measurements of triglyceride and total, low-density lipoprotein (LDL), and high-density lipoprotein (HDL) cholesterol concentrations measured by enzymatic methods (Roche Diagnostics GmbH, Mannheim, Germany). Serum creatinine was measured by an automated technique based on the measurement of Jaffe chromogen implemented in an auto-analyzer. Estimated glomerular filtration rate (e-GFR) was calculated using the equation proposed by investigators in the chronic kidney disease epidemiology (CKD-EPI) [27]. We preferred this equation because it is more accurate in subjects with a GFR > 60 mL/min/1.73 m^2^, which our patients were expected to have (creatinine value < 1.5 mg/dL). High-sensitivity *C*-reactive protein (hs-CRP) was measured by a turbidimetric immunoassay (Behring).

### Blood pressure measurement

After an initial blood pressure (BP) measurement in both arms to exclude a possible difference between them (less than 10 mmHg), estimation of clinic BP was achieved after 5 min of quiet rest. A minimum of three BP readings were taken on three separate occasions at least 2 weeks apart. Systolic (SBP) and diastolic (DBP) BP were measured by a standard validated sphygmomanometer at the first appearance (phase I) and the disappearance (phase *V*) of Korotkoff sounds. Baseline BP values represent the average of the last two of the three consecutive measurements obtained at intervals of 3 min. The diagnosis of hypertension was based on values of clinic SBP ≥ 140 and/or DBP ≥ 90 mmHg, respectively. Pulse pressure was defined as the difference between systolic and diastolic BP.

### Endothelial function evaluation

Strain gauge plethysmography was used for the evaluation of endothelial function using the method initially proposed by Panza [7], and subsequently adopted by our group [11, 18–20, 28–34], the details of which have been previously published [11].

Briefly, all studies, after overnight fasting, were performed at 9:00 A.M. in a quiet air-conditioned room (22–24 °C) by the same experienced investigators with the subject lying supine. Vascular reactivity was investigated by measuring forearm blood flow (FBF) and BP during intra-arterial infusion of saline, acetylcholine (ACh), and sodium nitroprusside (SNP) at increasing doses. Measurements of FBF and vascular resistance were repeated every 5 min until stable. Endothelium-dependent and endothelium-independent vasodilation were assessed by a dose–response curve to intra-arterial ACh infusions (7.5, 15, and 30 μg/mL/min, each for 5 min) and SNP infusions (0.8, 1.6, and 3.2 μg/mL/min, each for 5 min), respectively. The sequence of administration of ACh and SNP was randomized to avoid any bias related to the order of drug infusion. Forearm vascular resistance, expressed in arbitrary units (*U*), was calculated by dividing mean BP by FBF. For the present study, both maximal response to ACh and SNP were considered for statistical analysis.

### Follow-up and cardiovascular events

All patients, according to current guidelines, were treated to reduce clinic BP < 140/90 mmHg using standard lifestyle and pharmacological treatment [35]. Diuretics, β-blockers, ACE-inhibitors, calcium channel blockers, angiotensin II receptor antagonists, and α1-blockers were used alone or in various associations without significant differences between the groups. Antihypertensive drugs used in the study population are listed in Table 1.

**Table 1:** Baseline demographic and clinical characteristics of the study population

	All (*n* = 844)	Males (*n* = 451)	Females (*n* = 393)	*P*
Age, yrs	49.7 ± 10.6	49.3 ± 10.5	50.1 ± 10.7	*0.136*
Body mass index, *K*g/m^2^	27.2 ± 3.4	27.0 ± 3.1	27.5 ± 3.8	*0.056*
Current smokers, %	192 (22.7)	104 (23.1)	88 (22.4)	*0.817*
Heart rate, bpm	72.5 ± 9.7	72.0 ± 9.8	72.9 ± 9.5	*0.115*
Systolic BP, mmHg	151.0 ± 16.2	151.0 ± 15.7	150.6 ± 16.7	*0.325*
Diastolic BP, mmHg	91.6 ± 10.9	91.8 ± 10.9	91.1 ± 11.0	*0.145*
Pulse pressure, mmHg	59.4 ± 12.2	59.2 ± 12.5	59.5 ± 12.0	*0.361*
Total cholesterol, mg/dL	204.5 ± 30.5	205.7 ± 31.0	205.3 ± 29.8	*0.391*
LDL-cholesterol, mg/dL	127.9 ± 31.7	126.8 ± 32.0	130.3 ± 31.4	*0.035*
HDL-cholesterol, mg/dL	50.1 ± 11.4	50.8 ± 11.2	49.2 ± 11.6	*0.008*
Triglyceride, mg/dL	118.8 ± 38.7	116.7 ± 38.0	120.9 ± 39.4	*0.344*
Fasting glucose, mg/dL	95.4 ± 10.1	95.5 ± 9.7	95.4 ± 10.4	*0.248*
Insulin, U/L	14.4 ± 5.9	13.9 ± 5.0	15.1 ± 6.7	*0.171*
HOMA	3.4 + 1.6	3.3 ± 1.3	3.6 ± 1.8	*0.175*
Creatinine, mg/dL	0.96 ± 0.19	0.95 ± 0.19	0.96 ± 0.20	*0.062*
e-GFR, mL/min/1.73 m^2^	83.5 ± 22.3	91.1 ± 21.2	76.6 ± 20.7	*0.0001*
hs-CRP, mg/L	3.8 ± 1.3	3.6 ± 1.3	4.0 ± 1.3	*0.031*
*Forearm blood flow*				
Basal, mL^.^100 mL tissue^−1^ min^−1^ ACh, % increase from basal SNP, % increase from basal	3.35 ± 0.66 293 ± 117 332 ± 102	3.31 ± 0.62 309 ± 108 333 ± 99	3.41 + 0.70 268 ± 113 327 ± 106	0.080 0.001 0.198
*Antihypertensive drugs*				
ACE-i/ARBs, *n* (%) Calcium antagonists, *n* (%) β-Blockers, *n* (%) α-Blockers, *n* (%) Diuretics, *n* (%) Associations, *n* (%)	673 (79.7) 291 (34.5) 49 (5.8) 15 (1.8) 164 (19.4) 509 (60.3)	356 (78.9) 154 (34.1) 26 (5.7) 9 (1.9) 86 (19.1) 270 (59.8)	317 (80.6) 137 (34.8) 23 (5.8) 6 (1.5) 78 (19.8) 239 (60.8)	0.533 0.827 0.956 0.607 0.775 0.779

*ACE-i* angiotensin-converting enzyme inhibitors, *ARBs* angiotensin II receptor blockers

The italics is referred to the unit of measure

During the follow-up, we planned periodic clinical controls, and a questionnaire was sent to family physicians. For the validation of clinical events, a local committee that operated based on source data (hospital records, death certificates or other original documents) was identified. In this analysis, we considered the following clinical events: fatal and non-fatal myocardial infarction (MI), and unstable angina, coronary revascularization procedures (percutaneous interventions and bypass graft surgery), cardiovascular death (aortic diseases underlying dissection or rupture, pulmonary embolism or edema) or death for any cause; fatal and non-fatal stroke and MACE (major adverse cardiovascular events) defined as MI, coronary revascularization procedures, stroke, cardiovascular death or for any cause. Diagnosis of acute MI was defined according to the criteria of the European Society of Cardiology/American College of Cardiology Foundation/American Heart Association/World Heart Federation [36]. Stroke was defined as a new neurological deficit of sudden onset that persisted for at least 24 h [37]. Subjects free from cardiovascular events were defined controls, while the other patients were considered cases.

### Statistical analysis

Data of this study were analyzed by standard descriptive and comparative tests. Results are reported as mean ± standard deviation (SD), and differences between clinical and biological data were tested by the unpaired Student’s *t* test and the Chi-square test for categorical variables as appropriated. Both ACh-mediated and SNP-mediated vasodilation were compared by analysis of variance (ANOVA) for repeated measurements and, when analysis was significant, we applied the Tukey test.

The association between FBF and the subsequent risk of outcome events was investigated by univariate and multivariate stepwise Cox regression analyses. The analysis of the effect modification of gender (1 male, 2 female) on endothelial function (maximal vasodilatory response to ACh) on the hazard ratio (HR) of the incidence rate of clinical events (MACE, coronary and cerebrovascular events and mortality) was investigated as suggested by Altman [38], by simultaneously including gender, ACh, and gender*ACh into the same multivariate model.

Tested covariates included ACh-stimulated FBF peak percent increase and several other cardiovascular risk factors, namely: gender, age, BMI, smoking, total and LDL cholesterol, triglyceride, SBP, HOMA and e-GFR. To avoid a possible co-linearity with e-GFR, we excluded creatinine as well as both fasting glucose and insulin from the analysis to avoid a possible collinearity with HOMA. In Cox models, data were expressed as HR, 95% confidence interval (95% CI), and *P* value.

Event rate is reported as the number of events/100 patient-years based on the ratio of the number of events observed to the total number of patient-years of exposure up to the terminating event or censor. For patients who are events free, the date of censor was that of the last contact. For the patients who experienced multiple events, survival analysis was restricted to the first event. Survival curves were estimated by use of the Kaplan–Meier product-limit method and compared using the Mantel log-rank test.

Receiver operating characteristic (ROC) analysis was used to compare the predictive validity and to determine the optimal cut-off values of peak percent increase in ACh-stimulated FBF. Area under the curve (AUC) was also measured to determine the diagnostic power of the test and to describe the probability that the peak percent increase in ACh-stimulated FBF values would correctly identify the subjects at risk of cardiovascular events.

Significant differences were assumed to be at *P* < 0.05. All calculations were done by SPSS for windows version 20, Chicago, Illinois, USA.

## Results

### Study population

Demographic, clinic, and biochemical characteristics of the study population stratified by sex [451 males (53.4%) and 393 females (46.6%)] are reported in Table 1. The mean age of the whole population was 49.7 ± 10.6 years, and there were 192 (22.7%) smokers.

**Fig. 1:**
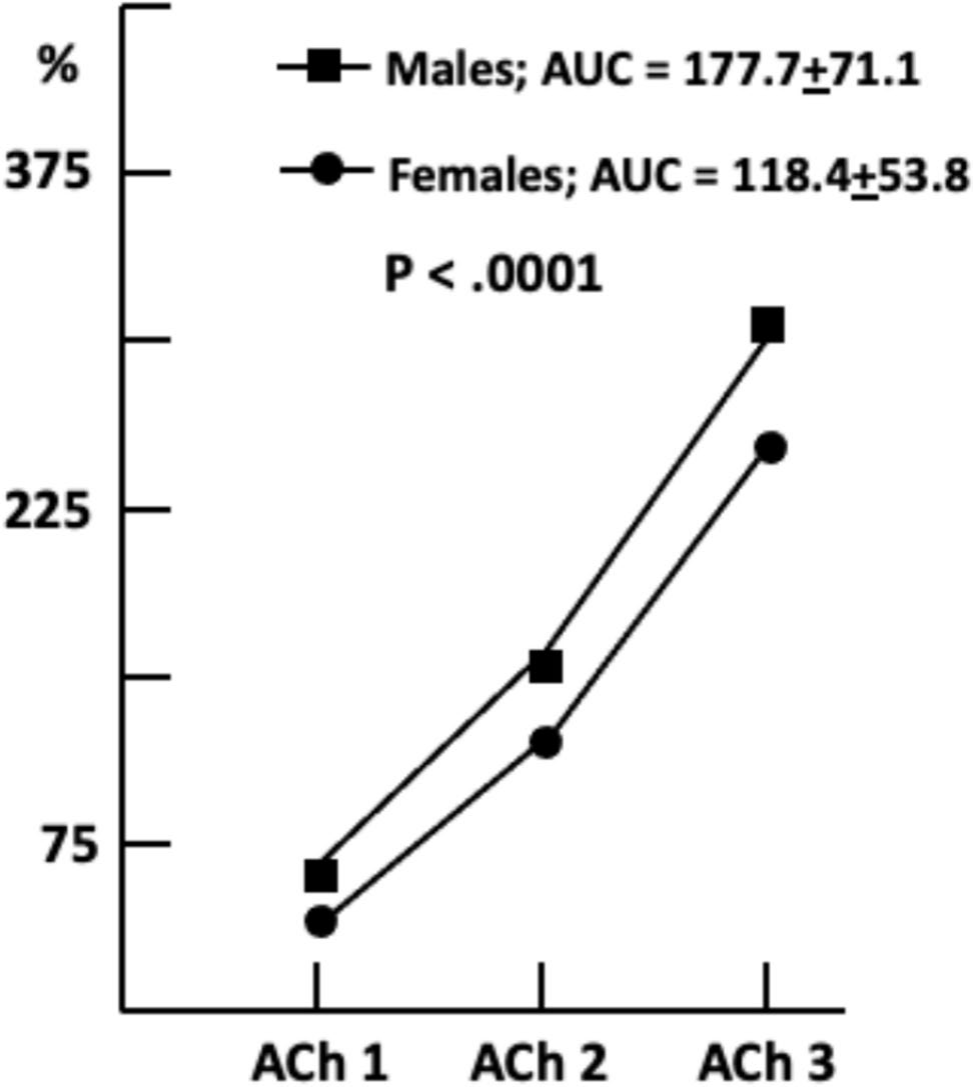
In the figure, the dose–response curves of forearm blood flow in both males and females during intravascular infusion of acetylcholine (ACh) are graphically reported. It is clearly evident, according to area under the curve (AUC), that females have a lower and significant endothelium-dependent vasodilation

There were no significant differences in age, BMI, smokers, heart rate, BP, total cholesterol, triglyceride, fasting glucose and insulin, HOMA, and creatinine between males and females. On the contrary, compared to males, females showed higher levels of LDL-cholesterol (130.3 ± 31.4 vs 126.8 ± 32.0 mg/dL, *P* = 0.035) and hs-CRP (4.0 ± 1.3 vs 3.6 ± 1.3 mg/L, *P* = 0.031), and significantly lower values of HDL-cholesterol (49.2 ± 11.6 vs 50.8 ± 11.2 mg/dL, *P* = 0.008) and e-GFR (76.6 ± 20.7 vs 91.1 ± 21.2 mL/min/1.73 m^2^). Finally, when considering forearm hemodynamic parameters, we did not observe significant differences in basal FBF and endothelial-independent vasodilation between groups. Of note, females showed a significantly lower endothelium-dependent vasodilation than males (ACh-stimulated peak percent increase 268 ± 113 vs 309 ± 108%, respectively). In Fig. 1, we graphically reported the dose-dependent curve during incremental dose of ACh in the females and males group; as evident, the area under the curve (AUC) is significantly lower (*P* < 0.0001) in females in comparison with males.

### Cardiovascular outcomes

**Fig. 2:**
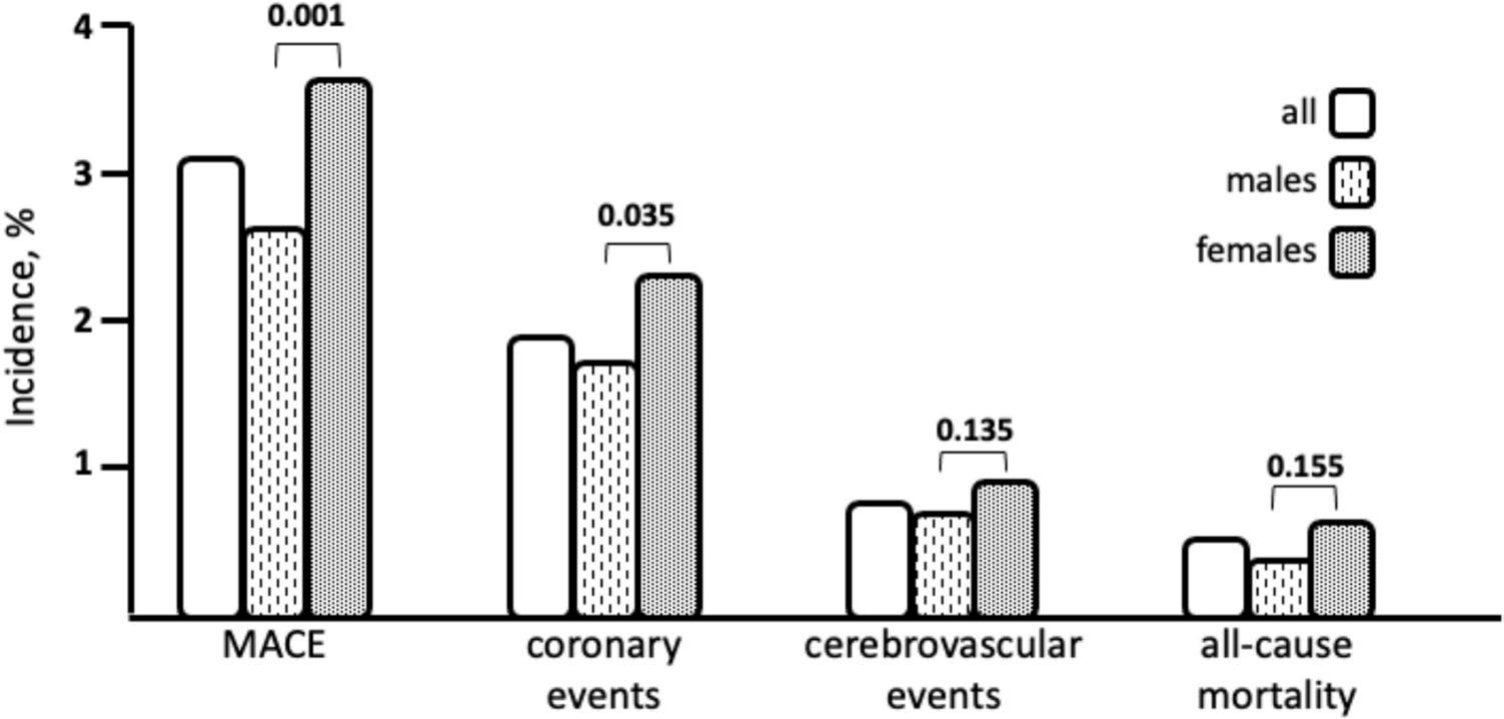
In this figure, the incidence of cardiovascular events is graphically reported in the whole study population and in the gender groups separately. As evident, the incidence of all cardiovascular events is higher in females in comparison to males

During the follow-up of 9.3 ± 3.2 years, we documented 252 new fatal (31) and non-fatal (252) cardiovascular outcomes (3.20%): 151 coronary (1.92%), 61 (0.77%) cerebrovascular, and 40 (0.51%) deaths (Table 2). Comparison between groups demonstrated that there was a significant difference between females and males regarding the incidence of both MACE (3.79 vs 2.68%, *P* = 0.001) and coronary events (2.24 vs 1.69%, *P* = 0.035); while, with regard to cerebrovascular events (0.93 vs 0.64%, *P* = 0.135) and all-cause mortality (0.63 vs 0.40%; *P* = 0.155), statistical significance was not reached, although a higher incidence was observed in women than in males. The incidence rate of clinical events in the whole study population and in females and males separately is graphically reported in Fig. 2.

**Table 2:** Cardiovascular events in the whole study population and in males and females

	All (*n* = 844)	Males (*n* = 451)	Females (*n* = 393)	*P*
MACE, (%)	252 (3.20)	113 (2.68)	139 (3.79)	*0.001*
Coronary events, (%)	151 (1.92)	69 (1.69)	82 (2.24)	*0.035*
Cerebrovascular events, (%)	61 (0.77)	27 (0.64)	34 (0.93)	*0.135*
All-cause mortality, (%) Cardiovascular Non-cardiovascular	40 (0.51) 31 9	17 (0.40) 13 4	23 (0.63) 18 5	*0.155*

The italics is referred to the unit of measure

### Cox regression analyses

On univariate Cox regression analysis in the whole study population, ACh-stimulated peak percent increase was significantly associated with the incidence rate of all study outcomes (Table 3); in particular, a 100% increase in FBF significantly reduces all cardiovascular events by about 30%: MACE (HR = 0.70; 95% CI 0.62–0.78), coronary events (HR = 0.70; 95% CI 0.60–0.81), cerebrovascular events (HR = 0.69; 95% CI 0.55–0.87) and all-cause mortality (HR = 0.72; 95% CI 0.54–0.95). As evident, other independent covariates for all pre-specified clinical outcomes in the whole study population were e-GFR, hs-CRP, and HOMA, while age, LDL-cholesterol, and triglyceride were retained in predicting both MACE and coronary events. Of interest, with the exclusion of cerebrovascular events and overall mortality, another important and independent prognostic factor for subsequent cardiovascular events was the female sex, confirming the existence of a significant biological difference between men and females.

**Table 3:** Univariate Cox regression analysis for incident MACE, coronary events, ictus, and all-cause mortality

	MACE			Coronary events			Cerebrovascular events			All-cause mortality		
*All*												
	HR	95% CI	*P*	HR	95% CI	*P*	HR	95% CI	*P*	HR	95% CI	*P*
ACh, 100% increase	0.70	0.62–0.78	0.000	0.70	0.60–0.81	0.000	0.69	0.55–0.87	0.002	0.72	0.54–0.95	0.019
e-GFR, 10 mL/min/1.7 m^2^	0.80	0.75–0.85	0.000	0.83	0.77–0.90	0.000	0.84	0.74–0.95	0.004	0.59	0.48–0.72	0.000
hs-CRP, mg/L	1.59	1.46–1.74	0.000	1.62	1.44–1.82	0.000	1.50	1.25–1.80	0.000	1.68	1.33–2.11	0.000
Age, 10 yrs	1.53	1.36–1.73	0.000	1.57	1.34–1.84	0.000	1.62	1.26–2.08	0.000	−	−	−
Gender, females vs males	1.50	1.17–1.52	0.002	1.44	1.04–1.99	0.027	−	−	−	−	−	−
HOMA	1.18	1.11–1.25	0.000	1.16	1.07–1.25	0.000	1.24	1.11–1.38	0.000	1.17	1.02–1.35	.027
LDL-Cholest, 10 mg/dL	1.12	1.08–1.16	0.000	1.15	1.10–1.22	0.000	−	−	−	−	−	−
Triglyceride, 10 mg/dL	1.07	1.03–1.10	0.000	1.07	1.03–1.11	0.001	−	−	−	1.11	1.02–1.20	0.012
BMI, *K*g/m^2^	1.07	1.04–1.11	0.000	−	−	−						
*Males*												
ACh, 100% increase	0.68	0.57–0.80	0.000	0.60	0.48–0.75	0.000	−	−	−	−	−	−
e-GFR, 10 mL/min/1.7 m^2^	0.81	0.74–0.88	0.000	0.83	0.75–0.93	0.001	v	−	−	0.56	0.42–0.75	0.000
hs-CRP, mg/L	1.93	1.66–2.25	0.000	2.18	1.78–2.66	0.000	1.52	1.13–2.06	0.006	1.90	1.28–2.82	0.002
Age, 10 yrs	1.04	1.02–1.06	0.000	1.04	1.02–1.06	0.000	1.04	1.01–1.08	0.015	−	−	−
HOMA	1.32	1.16–1.50	0.000	1.26	1.06–1.50	0.008	1.38	1.05–1.80	0.020	1.46	1.08–1.96	0.014
LDL-Cholest, 10 mg/dL	1.11	1.05–1.18	0.000	1.12	1.04–1.21	0.003	−	−	−	−	−	−
Triglyceride, 10 mg/dL	1.08	1.03–1.14	0.001	1.08	1.01–1.15	0.020	−	−	−	−	−	−
*Females*												
ACh, 100% increase	0.67	0.58–0.78	0.000	0.72	0.60–0.87	0.000	0.60	0.44–0.81	0.001	−	−	−
e-GFR, 10 mL/min/1.7 m^2^	0.78	0.71–0.86	0.000	0.82	0.73–0.92	0.001	0.78	0.64–0.93	0.007	0.61	0.45–0.81	0.001
hs-CRP, mg/L	1.46	1.29–1.64	0.000	1.39	1.19–1.62	0.000	1.51	1.19–1.91	0.001	1.57	1.16–2.11	0.003
Age, 10 yrs	1.05	1.03–1.07	0.000	1.05	1.03–1.08	0.000	1.05	1.02–1.09	0.004	−	−	−
HOMA	1.16	1.08–1.24	0.000	1.16	1.06–1.26	0.001	1.23	1.08–1.39	0.001	−	−	−
LDL-Cholest, 10 mg/dL	1.13	1.07–1.19	0.000	1.19	1.11–1.27	0.000	−	−	−	−	−	−
Triglyceride, 10 mg/dL	1.06	1.01–1,10	0.002	1.06	1.01–1.12	0.027	−	−	−	−	−	−

After the preliminary multiple Cox regression model, by which we tested the possible interaction between gender and ACh-stimulated FBF, the subsequent multivariate Cox regression analysis (Table 4) including all covariates retained as significantly in the univariate models, we observed that ACh-stimulated FFB, in the whole population, remained significantly associated with the occurrence of MACE (HR = 0.82; 95% CI 0.73–0.952) and coronary events (HR = 0.81; 95% CI 0.69–0.95), together with hs-CRP, age, LDL-cholesterol, and triglyceride. The same analysis also showed that FBF peak percent increase was retained as an independent predictor, together with other covariates as reported in Table 4, in both males (HR = 0.73; 95% CI 0.61–0.87) and females (HR = 0.81; 95% CI 0.68–0.97). Moreover, endothelial-dependent vasodilatation was retained, only in women, as an independent and significant predictor of cerebrovascular events (HR = 0.74; 95% CI 0.52–0.93). In this context, the comparison of the incidence of clinical outcomes demonstrated that females, compared to males, have an increased risk (+ 41.4%) for the occurrence of MACE and coronary events (+ 45.3%).

**Table 4:** Multivariate Cox regression analysis for incident MACE, coronary events, ictus, and all-cause mortality

	MACE			Coronary events			Cerebrovascular events			All-cause mortality		
*All*	HR	95% CI	*P*	HR	95% CI	*P*	HR	95% CI	*P*	HR	95% CI	*P*
ACh, 100% increase	0.82	0.73–0.92	0.001	0.81	0.69–0.95	0.012	−	−	−	−	−	−
e-GFR, 10 mL/min/1.7 m^2^	0.89	0.84–0.95	0.001	−	−	−	−	−	−	0.62	0.51–0.76	0.000
hs-CRP, mg/L	1.40	1.27–1.54	0.000	1.42	1.25–1.61	0.000	1.34	1.10–1.64	0.004	1.41	1.10–1.82	0.008
Age, 10 yrs	1.25	1.09–1.44	0.002	1.34	1.11–1.60	0.002	1.37	1.04–1.82	0.028	−	−	−
Triglyceride, 10 mg/dL	1.05	1.02–1.08	0.002	1.05	1.01–1.09	0.016	−	−	−	1.09	1.01–1.18	0.012
LDL-Cholest, 10 mg/dL	1.06	1.02–1.10	0.027	1.10	1.04–1.16	0.000	−	−	−	−	−	
*Males*												
ACh, 100% increase	0.73	0.61–0.87	0.000	0.64	0.50–0.81	0.000	−	−	−	−	−	−
e-GFR, 10 mL/min/1.7 m^2^	0.87	0.79–0.96	0.008	−	−	−	−	−	−	−	−	−
hs-CRP, mg/L	1.63	1.37–1.94	0.000	1.90	1.52–2.39	0.000	−	−	−	−	−	−
Triglyceride, 10 mg/dL	1.07	1.02–1.12	0.005	−	−	−	−	−	−	−	−	−
*Females*												
ACh, 100% increase	0.81	0.68–0.97	0.024	0.83	0.74–0.95	0.000	0.74	0.52–0.93	0.002	−	−	−
e-GFR, 10 mL/min/1.7 m^2^	0.87	0.79–0.96	0.006	−	−	−	−	−	−	0.64	0.48–0.86	0.003
hs-CRP, mg/L	1.29	1.13–1.46	0.000	1.22	1.04–1.45	0.016	1.35	1.04–1.76	0.026	1.44	1.03–2.01	0.034
Age, 10 yrs	1.03	1.01–1.05	0.008	1.04	1.01–1.06	0.006	−	−	−	−	−	−
LDL-Cholest, 10 mg/dL	1.07	1.01–1.13	0.025	1.15	1.06–1.24	0.001						

### ROC analysis

**Fig. 3:**
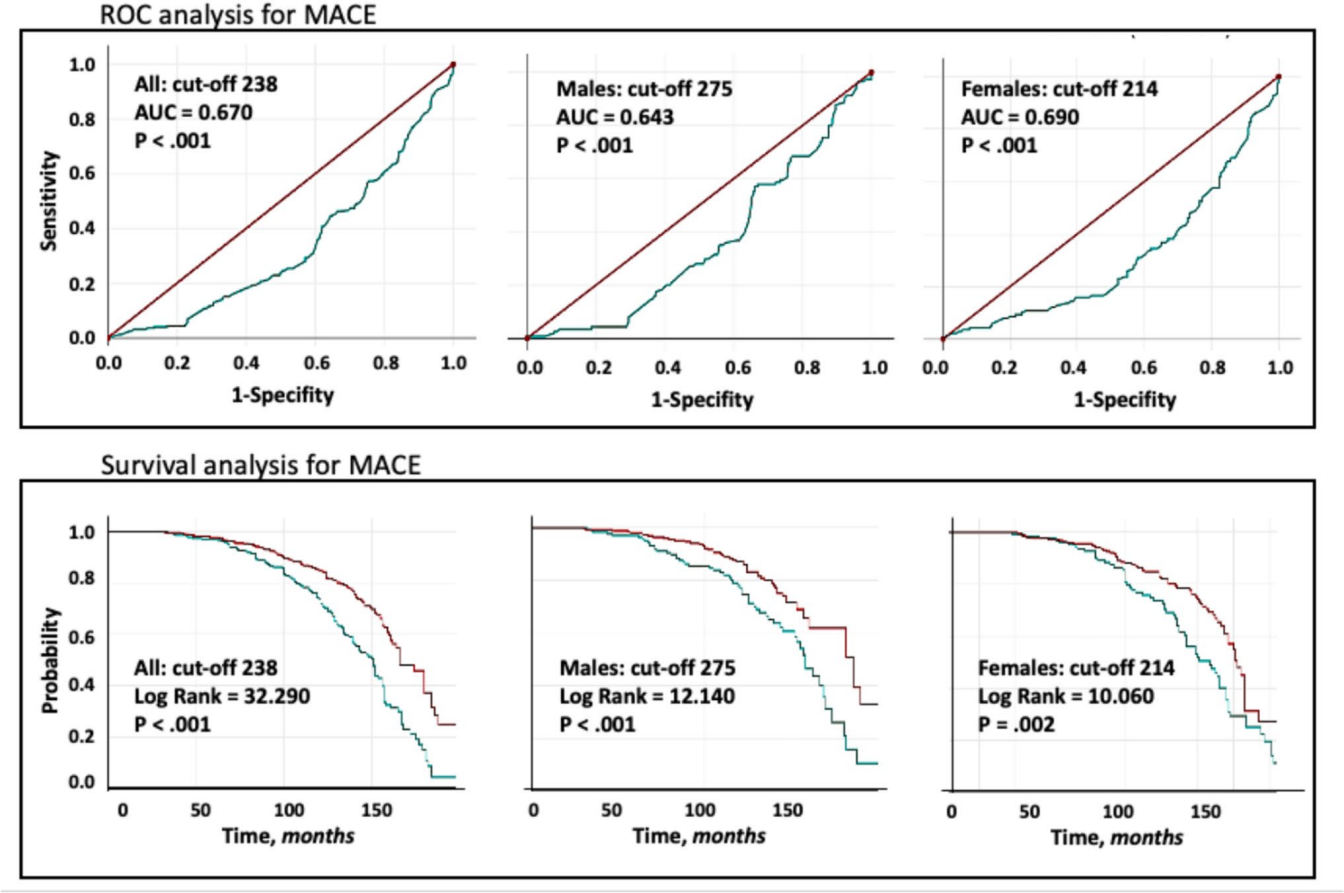
Results of both ROC and survival analyses are reported. It is evident that the best cut-off, in addition to being different between males and females (275 vs 214), can significantly discriminate the Kaplan–Meier curves

In Fig. 3, we reported the ROC curves for regression-fitted values of peak percent increase in ACh-stimulated FBF in predicting MACE in the whole population and in both female and male groups. Although endothelial function predicts MACE occurrence equally well in both groups, it is evident that the best cut-off value is lower in females than in males, confirming the possible pathogenetic role of vascular damage in the occurrence of cardiovascular outcomes.

In the same figure are reported the Kaplan–Meier survival curves for MACE in the whole population and in women and men separately, divided into above and under best cut-off of peak percent increase in ACh-stimulated FBF, that is 275% for males and 214% for females.

## Discussion

Results of this study demonstrate that endothelium-mediated vasodilation, evaluated by strain-gauge plethysmography in a large and well-characterized group of newly diagnosed hypertensive patients, is significantly reduced in females compared to males. This evidence has greater biological plausibility because the two groups do not differ in metabolic and hemodynamic parameters associated with endothelial dysfunction [7–9, 18–20, 28–34]. This different vasodilatory response observed in our population can be explained by a greater inflammatory status present in the female group, as highlighted by higher levels of hs-CRP which is well demonstrated to be associated with endothelial dysfunction [10, 12, 15–19, 29–34]. With regards to this point, however, it should not be forgotten that females can have systemic and coronary hemodynamics different from that of males as a consequence of structural and functional vascular wall characteristics between the two sexes [38]. Thus, it is possible that these vascular differences may change blood velocity and, therefore, modify shear stress from laminar to oscillatory which, as known, exert less protective anti-atherosclerotic vascular effects [15–18, 40–44].

Of great interest and in agreement with this evidence, a statistically significant higher incidence of clinical events was observed in the female group with regards to MACE and coronary events. According to this, in the multivariate Cox analysis, endothelial dysfunction was retained, together with other covariates, as a strong and independent predictor of MACE and coronary events in males and of MACE, coronary, and cerebrovascular events in females. Atherosclerotic cardiovascular diseases remain the leading cause of fatal and nonfatal cardiovascular events, especially in industrialized and developing countries, with a less favorable impact, in terms of prognosis and therapy, in women than in males [1–4]. It is well demonstrated that atherosclerotic vascular disease is the main pathogenetic mechanism involved in both cardiovascular morbidity and mortality. Therefore, to implement an effective and efficient preventive strategy, it is necessary to identify subjects with cardiometabolic risk factors and/or the presence of subclinical organ damage from its initial stages as early as possible to improve lifestyle modifications and enhance the most appropriate pharmacological treatment.

Thus, according to this, it is particularly important to early recognize atherosclerotic vascular damage from its initial stage, such as endothelial dysfunction. In fact, it is clearly established that endothelial dysfunction, characterized by a blunted vasodilating response due to reduced nitric oxide (NO) bioavailability, is an independent and strong predictor of cardiovascular events as initially demonstrated by us [11] and, successively, confirmed by others [12]. The biological plausibility of this is supported by the fact that the disruption of normal endothelial function actively participates in all pathogenetic mechanisms involved in the appearance and progression of the atherosclerotic process of all vascular districts, including the coronary one [28, 30–32]. Furthermore, the prognostic significance of endothelial dysfunction in the pathogenesis of clinical events is supported by its active participation in the destabilization of the atherosclerotic plaque through the modification of coronary shear stress and activation of some metalloproteinases mediated by systemic and local inflammation [40–44]. According to this, in our population, we observed a higher significant increase in hs-CRP in females when compared with males; in addition, hs-CRP was retained as a strong and significant predictor of all cardiovascular outcomes in both sexes.

Our data also show that females have significantly lower e-GFR values (about 15 mL/min/1.73 m^2^) than men, independent of BP value and age. It is not unlikely that this renal impairment may have contributed to the excess risk observed in females, as it is widely known that renal damage is associated with a higher cardiovascular morbidity and mortality in the general population as well as in different settings of patients [45, 46]. On the other hand, it is important to remember that previously published findings also demonstrated an interesting association between endothelial dysfunction and renal failure [30, 31, 34]. In this context, we clearly demonstrated that low-grade inflammation, evaluated by hs-CRP measurement, was the common pathogenetic mechanism mediating renal insufficiency and endothelial dysfunction [30, 31, 33]; the same mild inflammation that is considered a strong and independent marker of atherosclerotic cardiovascular morbidity and mortality and stratification of residual cardiovascular risk [47–49]; interestingly, this residual inflammatory risk appears to be more strongly associated with future cardiovascular events than residual cholesterol risk.

## Conclusion

In conclusion, our results confirm the presence of a significant difference between females and males in the occurrence of both fatal and non-fatal atherosclerotic cardiovascular events in patients with hypertension. Clinically relevant, this difference seems to be attributable, with good biological plausibility, to a lower vasodilating property of vascular endothelium observed in the females. Thus, according to this, it is important to remember, in the stratification of cardiovascular risk, that the vascular system of females may have morphological and functional characteristics different from those of males. Finally, it is desirable that our results will help to debunk the concept that females are to be considered at low cardiovascular risk, considering atherosclerotic cardiovascular diseases as a clinical condition predominantly impacting females [39]. For all these reasons, it is likely that females have received less attention to the diagnosis and treatment of cardiovascular risk factors, affecting a very high incidence of fatal and non-fatal cardiovascular events, especially in the post-menopausal period.

## Data Availability

The datasets generated during and/or analysed during the current study are available from the corresponding author on reasonable request.
